# An ultrahigh-accuracy Miniature Dew Point Sensor based on an Integrated Photonics Platform

**DOI:** 10.1038/srep29672

**Published:** 2016-07-15

**Authors:** Jifang Tao, Yu Luo, Li Wang, Hong Cai, Tao Sun, Junfeng Song, Hui Liu, Yuandong Gu

**Affiliations:** 1 Institute of Microelectronics, A^*^STAR (Agency for Science, Technology and Research), 11 Science Park Road 117685, Singapore; 2 School of Electrical and Electronic Engineering, Nanyang Technological University, 50 Nanyang Avenue 639798, Singapore; 3 National Metrology Center, A^*^STAR (Agency for Science, Technology and Research), 1 Science Park Drive 118221, Singapore

## Abstract

The dew point is the temperature at which vapour begins to condense out of the gaseous phase. The deterministic relationship between the dew point and humidity is the basis for the industry-standard “chilled-mirror” dew point hygrometers used for highly accurate humidity measurements, which are essential for a broad range of industrial and metrological applications. However, these instruments have several limitations, such as high cost, large size and slow response. In this report, we demonstrate a compact, integrated photonic dew point sensor (DPS) that features high accuracy, a small footprint, and fast response. The fundamental component of this DPS is a partially exposed photonic micro-ring resonator, which serves two functions simultaneously: 1) sensing the condensed water droplets via evanescent fields and 2) functioning as a highly accurate, *in situ* temperature sensor based on the thermo-optic effect (TOE). This device virtually eliminates most of the temperature-related errors that affect conventional “chilled-mirror” hygrometers. Moreover, this DPS outperforms conventional “chilled-mirror” hygrometers with respect to size, cost and response time, paving the way for on-chip dew point detection and extension to applications for which the conventional technology is unsuitable because of size, cost, and other constraints.

The precise, real-time measurement of relative humidity (RH) is highly important for applications ranging from appropriately correcting for the density of the air in standardized conditions for aero engine acoustic testing to accurate natural gas metering^1^,^2^,^3^,^4^,^5^,^6^,^7^,^8^. Many types of humidity sensors or hygrometers have been developed over the years^9^,^10^,^11^,^12^,^13^,^14^,^15^,^16^. The common, cost-effective miniaturized solutions utilize a solid-state sensing film that interacts with water vapour and converts the water vapour concentration into an electrical signal^10^,^11^,^12^,^13^. These traditional electronic humidity sensors have the universal shortcomings of drift and contamination-induced inaccuracy, which typically limits their accuracy in the range of +/−1% to +/−5%; their accuracy is often even poorer in low RH (0~20%) and high RH (80~100%) environments^11^. For applications that require higher accuracy, in industry, “chilled-mirror” dew point hygrometers are used^14^,^15^,^16^,^17^; in these devices, a polished mirror is cooled by a thermoelectric cooler, and a platinum resistance thermometer (PRT) is imbedded within the mirror surface to measure its surface temperature, as shown in Fig. 1a. An electro-optic detection system, which consists of a light-emitting diode (LED) and a light detector, is used to monitor the fine water droplets that condense on the mirror surface by detecting the reduction in the reflected light resulting from droplet-induced scattering. Its accuracy for dew point temperature measurements is typically +/−0.1 °C over a wide range of temperatures^14^. However, its high price (up to US$50,000), large footprint (>100 cm

^3^), and slow response (minutes), particularly for lower dew point temperatures (i.e., up to several hours for a dew point temperature below −60 °C), limit its wide adoption. Achieving greater accuracy, however, is challenging because of the effects of many types of temperature errors, e.g., temperature error because of self-heating and measurement hysteresis of the embedded thermometer. Recently, some potentially disruptive solutions for high-accuracy detection, such as carbon nanotubes^18^,^19^ and graphene^20^−^22^, have been reported. Although these materials have shown very high humidity sensitivities, they likely suffer from contaminant-induced interference.

Integrated photonics technology has matured over recent decades and constitutes a miniaturized and cost-effective platform for on-chip sensing and communication^23^,^24^,^25^,^26^. Various photonic resonator-based sensors with very attractive performances have been developed. For example, a parts-per-billion level gas-phase chemical sensor has been successfully demonstrated based on a high-quality factor (Q-factor) silicon photonics crystal structure with a footprint of only several square micrometres^27^. In this paper, we report a novel dew point detection solution based on the integrated photonics platform. Compared to “chilled-mirror” hygrometers, this device features high accuracy, a small footprint, and fast response. Moreover, this cost-effective, mass-manufacturable photonic dew point sensor (DPS) eliminates the cost/performance trade-off that affects both electrical thin-film humidity sensors and “chilled-mirror” hygrometers.

## Design and Fabrication

**Figure 1:**
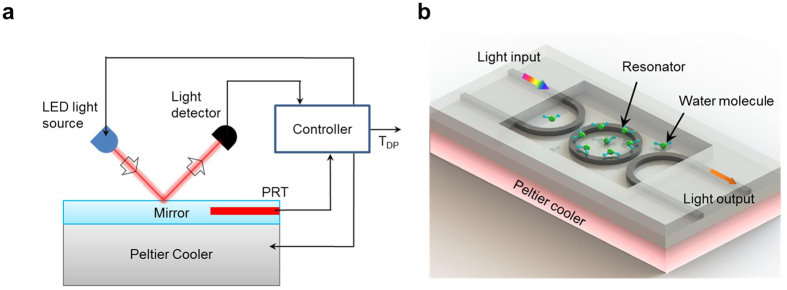
(**a**) Conventional “chilled-mirror” dew point hygrometer. (**b**) Integrated photonics DPS based on a photonics resonator cavity. The resonator acts as a “chilled-mirror” and a thermometer simultaneously.

The resonance frequencies/wavelengths of a high-Q-factor photonic resonator cavity are susceptible to variation in the dielectric properties of the surrounding medium. Hence, by monitoring the shifts of the resonance frequencies/wavelengths, the dielectric properties of the environment can be probed with very high accuracy. In this work, we report a general approach to designing an on-chip photonic micro-ring resonator for dew point temperature detection. The schematic of the device is shown in Fig. 1b. A partially exposed micro-ring resonator cavity chip is placed on top of a thermoelectric cooler, which cools the device to below the dew point temperature. The resonator supports high-Q whispering gallery modes, whose resonance frequencies are susceptible to variations in the dielectric environment^27^,^28^. In our design, the micro-ring resonator serves two functions: 1) probing the temperature change in the environment via a thermos-optic effect (TOE)-induced resonant wavelength shift^29^,^30^ and 2) detecting the water droplets condensed on the device via the evanescent wave of the light^31^,^32^. When the chip is cooled to the dew point temperature, water droplets condense on the top surface of the resonator. The condensed water droplets have a much higher refractive index than the air surrounding the micro-ring resonator before cooling, which causes a significant shift in the resonance wavelengths of the micro-ring resonator. By recording this wavelength change, the dew point temperature can be determined.

**Figure 2:**
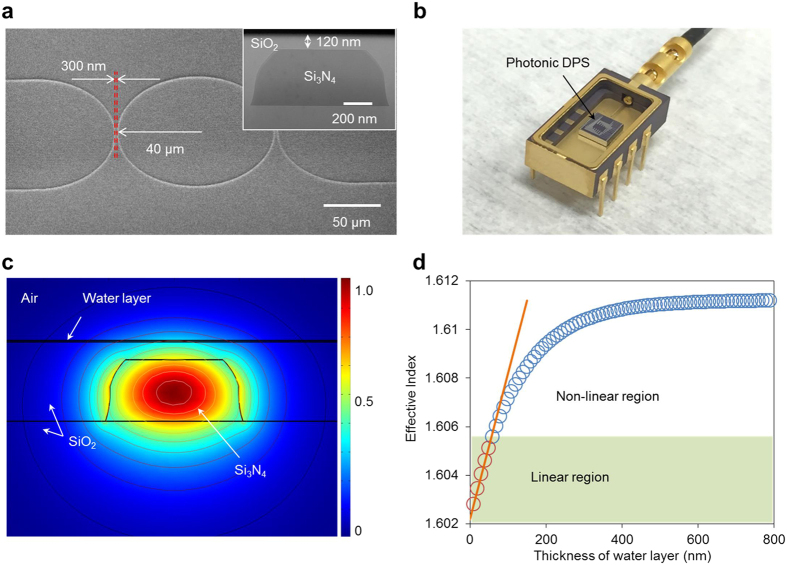
Micro-resonator images and simulations. **(a)** SEM micrograph of the Si_3_N_4_ resonator. Inset shows the cross-section of the Si_3_N_4_ waveguide structure (0.4 μm × 1 μm, Height × Width) with trimmed edges for low loss light propagation. A 120-nm thick SiO_2_ layer is placed on the Si_3_N_4_ waveguide to provide a pristine water condensation surface. **(b)** Packaged DPS. **(c)** Finite element simulation result showing the *E*_*x*_ component of the optical fields in the waveguide evanescently propagated in the air. The evanescent field ratio is approximately 4.1% in air. **(d)** Simulation of the effective index changes vs. the thickness of the water layer.

A silicon nitride (Si_3_N_4_) platform was used to fabricate the micro-ring resonator, which satisfies our low optical loss and high-Q requirements. Although the TOE coefficient of Si_3_N_4_ is less than that of silicon, its low refractive index enables a stronger evanescent field, improving its sensitivity to condensed water droplets^33^. Figure 2a shows a scanning electron microscope (SEM) micrograph of the Si_3_N_4_ resonator, which has a 40-μm diameter and a 300-nm coupling gap. The first steps in the fabrication are the deposition of a 3.5-μm-thick silicon dioxide (SiO_2_) layer and a 400-nm-thick Si_3_N_4_ layer in a low-pressure chemical vapour deposition (LPCVD) furnace, which is followed by the creation of waveguide structures defined using deep ultraviolet (UV) photolithography. Reactive ion etching (RIE) is used to transfer the waveguide patterns to the Si_3_N_4_ layer. A second RIE dry etch is performed to remove the waveguide edges and minimize the optical transmission loss. An 800-nm SiO_2_ cladding layer is then blanket deposited onto the entire device structure via plasma-enhanced chemical vapour deposition (PECVD). Finally, a chemical mechanical polishing (CMP) process is used to thin down the cladding SiO_2_ layer to 120 nm. This CMP process provides a pristine surface for water molecule condensation, as shown in the insert of Fig. 2a. The photonics chip is packaged via the advanced passive photonics packaging method, as we reported previously^25^ (Fig. 2b). A grating coupler is used to couple light into and out of the sensor. The grating coupler is designed with a period of 1.1 μm, a filling factor of 50%, and an etching depth of 400 nm. The minimum fibre-waveguide-fibre insertion loss of a waveguide with grating couplers is approximately −14.6 dB at a wavelength of approximately 1590 nm. To reduce the packaging procedure, a reflector based on a waveguide-loop mirror is used to reflect the resonant light back so that both the input and output light can share a common fibre^34^. With this structure, a Q-factor of 24,000 and a free spectral range (FSR) of 5.2 nm are achieved for the micro-ring resonator, and the waveguide transmission power loss is 0.36 dB/cm. The optical fields of the micro-ring resonator decay exponentially outside its geometric boundaries, as simulated in Fig. 2c. The energy of the evanescent wave accounts for approximately 4.1% of the total photonic energy. The effective index of the micro-ring resonator increases with the water layer thickness. According to theoretical calculation, the effective index of the water layer increases almost linearly with the thickness when the water layer thickness is below 50 nm, as indicated in Fig. 2d. A linear relationship between changes in the effective index (*N*_*eff*_) and the water layer thickness (*t*) was observed (*N*_*eff*_ = 6 × 10^−5^ *t* + 1.6022, *R*^*2*^ = 0.9969) for water layer thicknesses from 0 to 50 nm. This relationship constitutes the foundation of the linear fitting for subsequent dew point temperature extraction.

## Experimental Results

**Figure 3:**
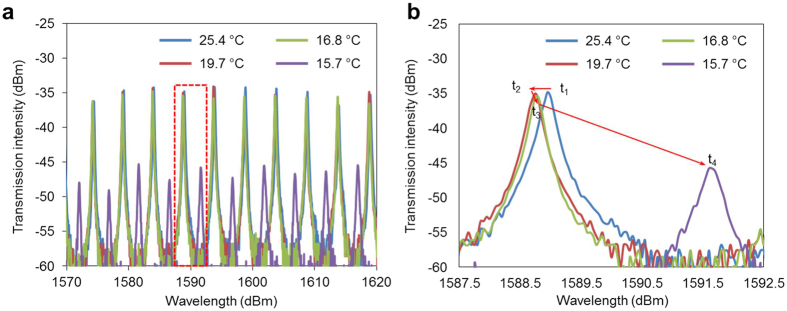
Transmission spectra of the ring resonator, measured without water condensation (above dew point 25.4 °C and 19.7 °C) and with water condensation (below view of the spectrum at approximately 1589 nm of Fig. 3a (red box).

Photonic characterization was conducted before subjecting the device to dew point testing in metrology laboratories. During the photonic characterization, the temperature of the device was controlled by a Peltier system controller (Model: Newport ILX Lightwave LDC-3700C), and the optical response was measured in the stationary state. A probe light from a broadband light source (Model: EXFO FLS-2300B) is coupled into the device through the grating coupler, and the output spectrum is monitored using an optical spectrometer (Model: ANDO AQ6317B). Figure 3a shows the transmission spectra of the micro-ring resonator at different temperatures. Figure 3b presents a magnified view of the spectrum shown in Fig. 3a at approximately 1589 nm (red box). Clearly, cooling the device induces a blue-shift of the resonant wavelengths when the device temperature is above the dew point (Fig. 3b, t_1_→t_2_). By contrast, when the device temperature is below the dew point, water starts to condense on the resonator, resulting in a drastic red-shift (Fig. 3b, t_3_→t_4_) that is observed because of the increased the refractive index of the resonator cladding layer (air→liquid water). Over the experimental temperature range (25.4 °C to 15.7 °C), the Q-factor of the resonant peak decreased from ~22,000 (blue and red lines, 25.4 °C and 19.7 °C, respectively) at room temperature to ~19,000 (green line, 16.8 °C) at the onset of condensation. Excessive cooling, which is manifested by extensive droplet coalescence, causes the Q-factor to decrease to values as low as ~6,000, largely because of light scattering from the large water droplets (purple line, 15.7 °C). Because the dew point is identified at the very onset of condensation, the Q-factor change is estimated to be less than 15% (from 22,000 to 19,000). This 15% Q-factor decease will cause the full width at half maximum (FWHM) to increase by 15%; thus, the resolution changes from 1 pm to 1.15 pm, and the dew point uncertainty is 0.004 °C (0.15 pm/(37 pm/°C)).

**Figure 4:**
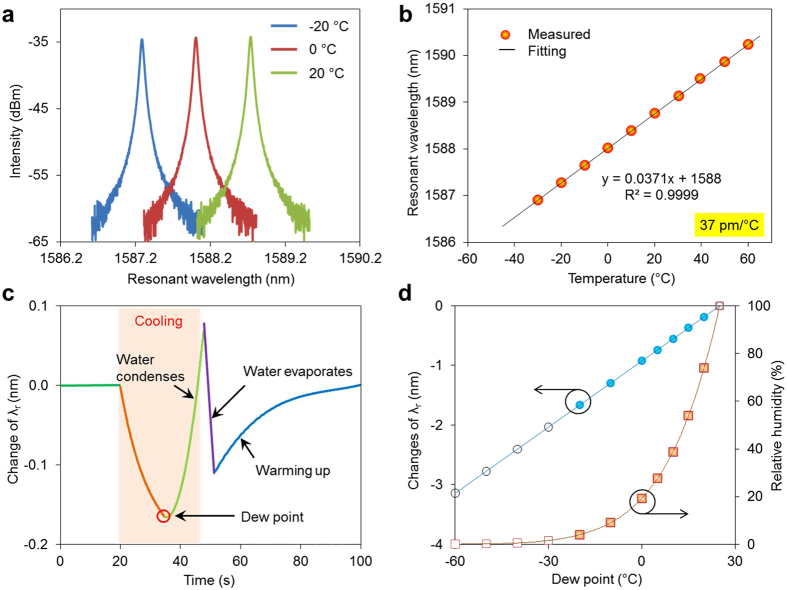
Spectra response of the sensing resonator. **(a)** Transmission spectral response to temperature change in terms of wavelength shift. **(b)** Near linear spectral shift in response to the temperature change, with a sensitivity of 37 pm/°C. **(c)** Typical wavelength response cycle of the resonator, from (TEC on) cooling without condensation and with condensation to (TEC off) water droplets evaporation and warming up. **(d)** Measured relationship between dew point temperature and RH vs. wavelength changes. Dew point temperature correlates linearly with resonant wavelength shift. RH is calculated based on dew point and ambient temperatures.

The photonic device was further characterized in a vacuum chamber (Model: Cascade PMV200) with a reference thermistor (US Sensor PR303J2). The resonator transmission spectra collected at different temperatures are shown in Fig. 4a,b. A linear spectral shift (*λ*) vs. temperature (*t*) was observed (*λ* = 0.0371 *t* + 1588, *R*^*2*^ = 0.9999) over the temperature range from −30 °C to 60 °C, with a sensitivity of ~37 pm/°C. Thus, a temperature resolution up to 0.006 °C is achievable with a 0.2 pm resolution wavelength meter. A temperature resolution of 0.006 °C is equivalent to a RH resolution of 0.0025% at 5% RH under ambient conditions. Our previous work demonstrated that the on-chip wavelength meter is fully capable of achieving such spectral resolution^35^.

Figure 4c presents a typical spectral response in dew point temperature detection relative to air sample with a prescribed dew point of 20.0 °C at an ambient temperature of 25 °C. In this experiment, a tuneable bandpass filter (Model: AlnairLabs BVF-200CL) was placed between the light source and the device. This tunable bandpass filter allows a narrow bandwidth light (~6 nm) couple into the device so that only one resonant peak generates at the device output. The resonator peak is monitored by a wavelength meter (Model: Burleigh WA-1600). Before the temperature of the chip reaches the dew point temperature, the resonance wavelength blue-shifted as the temperature decreased (orange line in Fig. 4c). However, when the sensor was cooled to the dew point temperature, water molecules started to condense on the surface of the chip patterned with the micro-ring resonator structure, eventually increasing the effective index of the micro-ring resonator structure abruptly because of water’s higher refractive index (*n*_*water*_ = 1.32, *n*_*air*_ = 1). Therefore, the resonance wavelength of the micro-ring resonator was red-shifted (green line in Fig. 4c). Fitting the data measured in these two temperature regimes separately generates an intersection, which indicates the temperature and time at which dew starts to form, i.e., the dew point temperature. The purple and dark blue lines indicate water droplet evaporation and the temperature returning to its original value, respectively. A more reliable measurement can be made by performing repeated cycles over a reasonably short time. Doing so eliminates the need for the temperature to return its original status, and the ambient temperature can be monitored using a separate resonator. Because of the unique features of the micro-ring resonator, the ambient temperature and dew point temperature can be extracted from the spectra. Thus, an external thermometer and complex temperature management system are not necessary.

To evaluate the device performance, dew point temperatures ranging from −20 °C to 20 °C were analysed. In the experimental setup, the photonic chip was placed in a metal chamber connected to a reference humidity generator (Thunder Scientific Model 4500) that generates nitrogen gas with known dew point temperatures. During the measurement, the gas passes through the metal chamber at a constant flow rate. The results are depicted by the blue circles in Fig. 4d. A good linear fitting line for the relationship between the dew point temperature and the resonant wavelength was obtained. Some forecasted resonant wavelengths are plotted using black open circles in Fig. 4(d). Note that photonic devices without a doping process are very stable at low temperatures^30^, indicating that a wider dew point temperature detection range can be achieved using a more powerful thermoelectric cooler. Based on the ambient temperature and dew point temperature measured by the photonic DPS, the corresponding RH value can be calculated directly using the formula^36^, as depicted by the orange squares in Fig. 4d. In a low-RH environment, the curve has a small slope, suggesting that a better resolution can be obtained; indeed, this is a challenging region for most miniaturized humidity sensors.

**Figure 5:**
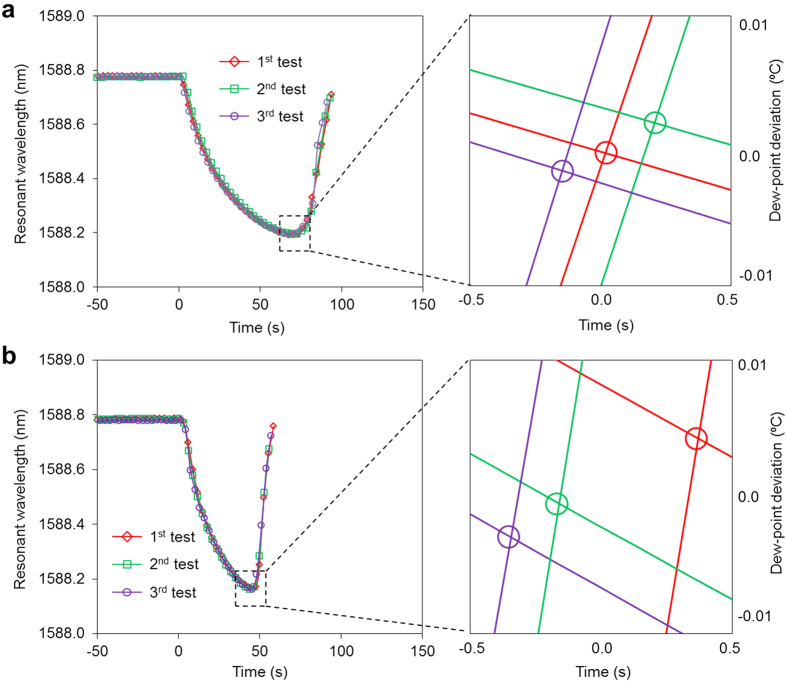
Repeatability testing of the proposed photonic DPS at different cooling currents. **(a,b)** are spectra responses at 5 °C dew point temperature using a thermoelectric cooler with a 1.0 A and 1.5 A supply current, respectively. The right hand figures show the magnified fitting lines. The close clustering of the extracted dew point temperatures (along the Y-axis) of repeated tests show 0.0022 °C and 0.0045 °C temperature deviations, respectively.

As discussed above, when the device is cooled to below the dew point temperature, the spectrum exhibits a blue-shift, followed by a red-shift as the environmental temperature decreases. Figure 5a,b show the test results for the same dew point temperature (5 °C) but different cooling currents (1 A and 1.5 A, respectively). Each condition was analysed three times. For each cooling current, the device exhibited very good testing repeatability, although the recording times differed by a few seconds. For the 1-A cooling current, the average wavelength at the intersection points (i.e., from blue-shift to red-shift) was 1588.156 nm, and the standard deviation was approximately 0.8 pm, which corresponds to a dew point temperature deviation of 0.022 °C. For the 1.5-A cooling current, the average wavelength at the intersection points was 1588.142 nm, and the standard deviation was approximately 1.7 pm, which is equal to a temperature deviation of 0.045 °C. In both cases, the data were selected according to the consistency of the linearity. Both deviations are believed to be mainly induced by the non-ideal behaviour of the water condensation process, which may include the unpredictable growth and merging of water droplets, which differs from the theoretical prediction, as presented in the “Design and Fabrication” section. In these two cooling tests, the average intersection wavelengths differed by 14 pm, which corresponds to a temperature difference of 0.38 °C. The main reason for this discrepancy was the slow sampling rate, which introduces an additional error in the fitting. This difference can be reduced using a high-speed photonics wavelength tracker instead of the conventional wavelength meter-based mechanical tuning approach^37^,^38^. An integrating tracker also contributes to reducing the total size and improving the accuracy of the photonic hygrometer.

## Discussion and Conclusions

“Chilled-mirror” dew point hygrometers suffer from intrinsic errors, which are mainly attributable to 1) the temperature gradient from the mirror surface to the embedded thermometer, 2) the thermal conductance of the thermometer leads, and 3) the self-heating of the thermometer and readout calibration^12^. These errors practically limit the resulting measurement accuracy to approximately +/−0.1 °C. Self-heating also occurs in photonics resonators and is a possible error source that may affect sensor accuracy^39^. Fortunately, this effect is relatively weak for the Si_3_N_4_ waveguide compared to silicon waveguides because the heating factor is negligible compared to two-photon absorption (TPA)^40^,^41^. For example, using a pump light of up to 200 mW, the self-heating effect has been shown to excite a wavelength shift of approximately 300 pm in a similar Si_3_N_4_ micro-ring resonator^41^. For a DPS, the operating power at the resonant wavelength is only ~0.002 mW (input power ~−19 dBm, coupling loss ~−7.3 dB for input), which is insufficient to generate an obvious self-heating effect in the sensor. The integrated photonic DPS reported here uses a single sensing element, i.e., a micro-ring resonator, to detect both the water condensation process and the corresponding temperatures, virtually integrating the “chilled-mirror” and thermometer together and eliminating the errors induced by the temperature gradient, thermal conductance and self-heating. As a result, it potential accuracy is greatly improved.

In summary, we developed a novel photonic DPS based on integrated photonics technology for highly sensitive dew point and RH detection by taking advantage of the thermo-optics effect and evanescent field sensing. Superior performance, including high accuracy and fast response, was achieved using a miniaturized silicon chip. The DPS demonstrated here provides a practical solution for accurate and reliable humidity measurements in the aerospace, meteorology, petrochemical, agriculture, biology, and pharmaceutics fields.

## Additional Information

**How to cite this article**: Tao, J. *et al*. An ultrahigh-accuracy Miniature Dew Point Sensor based on an Integrated Photonics Platform. *Sci. Rep.* **6**, 29672; doi: 10.1038/srep29672 (2016).
